# Community Response and Engagement During Extreme Water Events in Saskatchewan, Canada and Queensland, Australia

**DOI:** 10.1007/s00267-017-0944-y

**Published:** 2017-11-06

**Authors:** Dena W. McMartin, Alison J. Sammel, Katherine Arbuthnott

**Affiliations:** 1 Environmental Systems Engineering, Faculty of Engineering and Applied Science, University of Regina, 3737 Wascana Parkway, Regina, SK S4S 0A2 Canada; 2 School of Education and Professional Studies, Griffith University, Gold Coast, Australia; 3 Department of Psychology, Campion College, 3737 Wascana Parkway, Regina, SK S4S 0A2 Canada

**Keywords:** Community resiliency, Community vulnerability, Disaster management, Extreme water events, Flood

## Abstract

Technology alone cannot address the challenges of how societies, communities, and individuals understand water accessibility, water management, and water consumption, particularly under extreme conditions like floods and droughts. At the community level, people are increasingly aware challenges related to responses to and impacts of extreme water events. This research begins with an assessment of social and political capacities of communities in two Commonwealth jurisdictions, Queensland, Australia and Saskatchewan, Canada, in response to major flooding events. The research further reviews how such capacities impact community engagement to address and mitigate risks associated with extreme water events and provides evidence of key gaps in skills, understanding, and agency for addressing impacts at the community level. Secondary data were collected using template analysis to elucidate challenges associated with education (formal and informal), social and political capacity, community ability to respond appropriately, and formal government responses to extreme water events in these two jurisdictions. The results indicate that enhanced community engagement alongside elements of an empowerment model can provide avenues for identifying and addressing community vulnerability to negative impacts of flood and drought.

## Introduction

Each Saskatchewan, Canada and Queensland, Australia are Commonwealth jurisdictions that have experienced, and continue to experience, significant impacts due to largely unanticipated extreme water events (EWE), such as floods and droughts. EWE in these regions do not discriminate across relative wealth, do not avoid regions of high population density, and are often unanticipated despite the growing body of scientific and engineering decision-making protocols and literature intended to mitigate negative impacts of EWE. These protocols and discourses rarely address the socio-political, economic or cumulative effects of EWE, nor do they address the particularly thorny challenges of jurisdiction and planning for boundary waters flowing across state/province or national borders. It is also becoming apparent that science and technology alone cannot address challenges arising from EWE (Sammel and McMartin 2014).

The well-documented evidence of large-scale EWE highlights the significant damage floods and droughts can cause, and has led to improved awareness of localized occurrences and effects within the general public. As the occurrence of EWE changes in the frequency, duration, intensity and timing, their influence on both human and natural systems become less predictable, more risky, and more expensive (St. Jacques et al. 2014). This uncertainty is extremely challenging to communicate and plan for across communities. Adding to these challenges is the lack of local data on disaster risk mitigations or potential solutions for reducing (or eliminating) impacts of recent flood and drought events. In sum, there may be insufficient communication or existence of useful information from past and projected EWE to create and inform community education and response systems, and to develop programs that support change in behaviour, activities, and both human and physical constructs that could reduce vulnerability to water extremes and damage post-event. Diaz and Hurlbert (2014) suggest this knowledge is essential for reducing community vulnerability.

One approach to reducing community vulnerability is community empowerment. Empowerment is a process whereby citizens can gain control over the factors and decisions that shape their lives (Labonte and Laverack 2008). Community empowerment, then, offers people an opportunity to negotiate power to gain a measure of control or power over community resources and decision-making (Pulla and Bhushan 2015). Examples of empowerment models are well established in health care related to offering patients and patient advocates (family, friends) the responsibility and privilege of owning as much of their own health care experience and outcomes as possible. This is achieved by providing improved communication, consultation, and education to impart knowledge for competently navigating health systems (Lacey et al. 2015a, b). Within this article, results are viewed through the lenses of engaging and empowering communities to effectively and appropriately navigate the responses to and impacts of EWE is similarly based on access to and appropriate application of knowledge of local resources, capacities, and opportunities to effect positive change.

The information herein presents an analysis of patterns of community response and opportunities for constructive and proactive community engagement to reduce community vulnerability to EWE. The analysis includes a case study of how communities within Queensland, Australia and Saskatchewan, Canada are engaged by application of evidence and information provided in post-event task force reports and disaster response briefings that include both quantitative and qualitative data sets. This paper also identifies key factors that inform and limit community engagement (and individual capacity to appreciate or act on impacts) in response to EWE, especially in ways that necessitate personal responsibility. Broadly, the two regions in this research were chosen as they have similar education and political structures, and because each has recently experienced significant droughts and floods that created significant negative infrastructural and social impacts. More specifically, each region experienced recording breaking floods in 2011 pointing to opportunities to invest in reducing community vulnerability through engagement and education. Where possible, discussions of community empowerment, as evidenced by direct reference or context in secondary sources, are included.

## Foundations: Policies, Perceptions, and Holistic Understandings of EWE

The complexities of multiple political jurisdictions, as well as discontinuous and unshared hydrologic monitoring networks, raise significant challenges for mitigating potential negative impacts of EWE, such as droughts and flooding. From the perspectives of engineering and science related to water management and planning for hydrologic events, there are well-established practices and analytical processes to aid in the development of infrastructure, emergency and contingency plans, and watershed scale solutions in support of improving community response and resiliency to water-related extremes. However, resource management policies and plans based on scientific and technical knowledge of water have not always been successful at ensuring the water needs of people (Muro and Jeffrey 2008). With pressing concerns of social justice and climate change, water management and response issues must be understood within social, economic, political and environmental contexts. There are challenges on several fronts around this however, including communications of risk within and to communities of citizens and decision-makers, changes in climate that affect the utility and applicability of the historical data used to predict EWE, and perceptions of risk to life and property in response to flood and drought (Morgan et al. 2002; Slovic 2000).

The way in which communities, industries, and citizens perceive and respond to risk or occurrence of disaster is often unclear, disorganized, and ad hoc (Haasnoot et al. 2013; Vallero and Letcher 2012). Hydrologic events, in particular, seem to evoke responses that are not well coordinated and planned, nor do communities tend to learn, react, and affect change in response to such events. In the communication and recognition of risk, few people would choose to live in a home that has an annual risk of 1% of burning down, but these same people will choose to build (and rebuild) a home in an area with an annual risk of 1% of being damaged by flooding (Vallero and Letcher 2012; Murphy 2012). In addition to the documented poor intuitive understanding of probability (Hastie and Dawes 2001; Tversky and Koehler 1994), such inconsistencies are likely attributable to a combination of the availability heuristic (Tversky and Kahneman 1973) and evolutionary tendencies to focus on immediate spatial and temporal threats (Griskevicius et al. 2012; Stoknes 2015). As a result risks associated with EWE are less not always top of mind or necessarily memorable, even if they are equally probable to a home fire, for instance.

Haasnoot et al. (2013) recommend a stepwise approach to incorporating the risks associated with flood and drought into infrastructure development and community planning. The three key elements proposed include: (a) time-based scenarios of projections for climate change, economic development, societal change, (b) a method for evaluating potential outcomes based on combinations of these time-based scenario projections, and (c) a stepwise policy analysis Haasnoot et al. (2013). Wheater and Gober (2015) add that science must acknowledge community education and produce outputs that result from lessons learned, using an iterative and collaborative approach to management and response to water-related extremes. The science must further include communication plans across groups of water managers, policy makers, scientists and engineers, educators and community leaders (Vallero and Letcher 2012; Wheater and Gober 2015).

Due to its classification as the driest continent on the planet, the Australia government has committed resources and infrastructure to improve the resiliency of communities to recover from droughts. Because of these persistent challenges with availability of water, Australia has implemented some of the world’s leading drought management and drought response plans (Mount et al. 2016; Sayers et al. 2017). Ongoing development and innovation in municipal infrastructure (e.g., urban rain gardens that filter and store water for irrigation or human use) are leading edge in response to community vulnerabilities to a dearth of available and accessible water resources. To varying extents, communities are engaged—and sometimes empowered, with support from state and federal governments, to design and implement technologies and practices that address these vulnerabilities in a proactive and incremental manner. Where engagement moves into empowerment is noted through the specific applications and decision-making made at the community level in context of community-specific needs, requirements, and capacity to design, build, and operate technical responses and practices for addressing potential impacts of EWEs.

This is also similar in Canada. In response to the high impact Canadian Prairies drought of the “Dirty 30 s”, all levels of government engaged in drought management and resiliency schemes, including infrastructure development, land use changes, and adaptive practices such as domestic water restrictions and creation of shelterbelts (Corkal and Adkins 2008; Diaz and Hurlbert 2014; Hurlbert 2014; McLeman et al. 2014; Weaver and Gunton 1982). However, there are few recent schemes in response to projections that indicate high probability of more intense and frequent rainfall events coupled with prolonged periods of drought. The literature further notes that the majority of climate adaptation activities and land-use changes in communities and provincial governments continue to focus on drought, leaving communities less prepared for flood conditions (Bowering et al. 2014; Buckland and Rahman 1999; Haque 2000; Kevinsen et al. 2014; Measham et al. 2011). On the Canadian Prairies, flood management is complicated by the interprovincial and international nature of floods, requiring collaborative and consultative efforts to preserve and protect life, property and livelihoods across political boundaries (Buckland and Rahman 1999; Easter and Perry 2011; Haque 2000; Hearns et al. 2014).

There is a significant and demonstrable need to better link the science to policy and political decision-making (Haasnoot et al. 2013; Wheater and Gober 2015). The promotion of community-level engagement, generation of ideas and incorporation of solutions, and engagement of community leaders to effect bottom-up change in identifying, prioritizing, and implementing real-world water management solutions is foremost in establishing cultures of activity, responsibility, and possibility (Schnoor 2008). By generating broad public consensus and engagement in water management and understanding community vulnerability to the impacts of water-related extremes can be reduced.

By assessing the documented outcomes and post-event analyses of two significant floods in the selected study regions, we attempt to highlight the potential pathways toward and benefits of community engagement through improved education and communication systems. For communities to be engaged in proactive and responsible behaviours requires that trust be established and that general publics have ready access to information and updates about the hazards, community responses, and individual responsibilities during EWE.

## Research Approach

### Study Regions

**Fig. 1:**
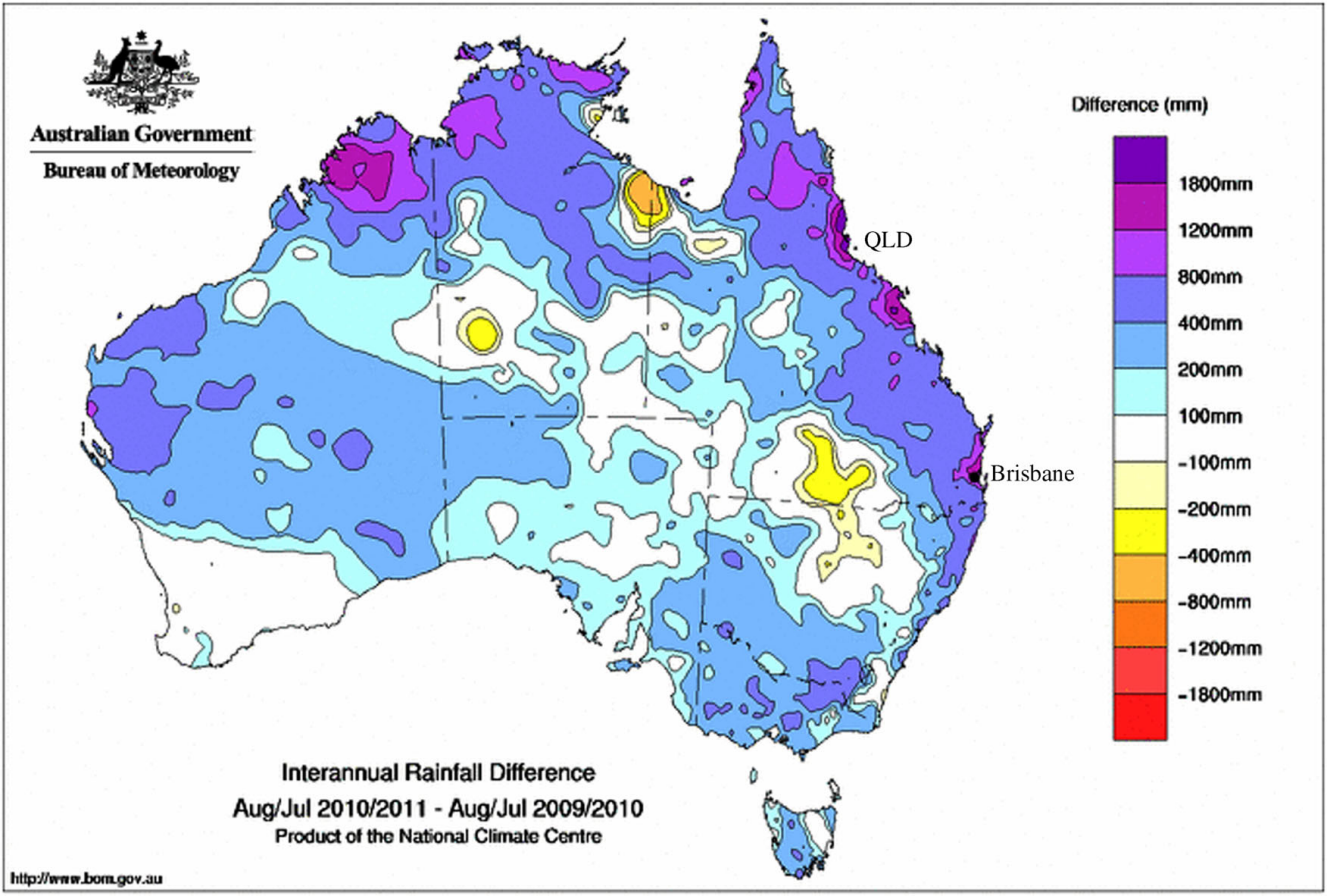
Queensland precipitation map for August 201 to July 2011 denoting the interannual rainfall difference across the country; note significantly higher than average rainfall along Queensland coastal regions in northeast Australia (Australian Bureau of Meteorology 2012).  Creative Commons Attribution Australia License

**Fig. 2:**
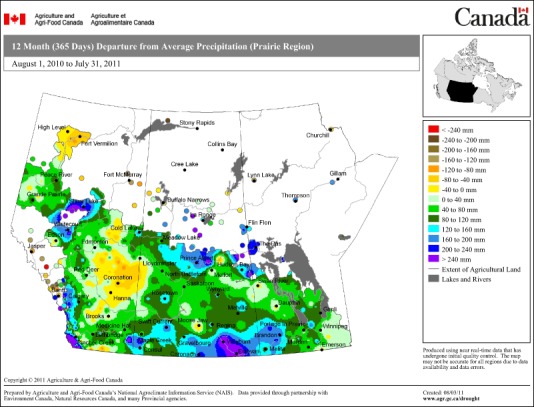
Canadian prairies region precipitation map for 1 August 2010 to 31 July 2011 denoting significant departures from average precipitation in southern regions affected by the 2011 EWE (Agriculture and Agri-Food Canada 2011). This map is a copy of an official work that is published by the Government of Canada and has not been produced in affiliation with, or with the endorsement of, the Government of Canada. Permission to reproduce

Each study region has experienced, and continues to experience, significant and largely unanticipated floods and droughts causing serious damage to property, communities, and social fabric. In Australia, the study region was constrained to the state of Queensland (QLD) (Fig. 1), focusing around the Brisbane area located along the coast in the south of the state. The Canadian event impacted portions of the three Prairie provinces (Fig. 2) and the northwestern Great Plains of the USA resulting in government documents being produced in multiple jurisdictions, each referencing the more narrowly scoped focus of this research being centred on the province of Saskatchewan.

The Australian study region lies very nearly on the boundary of the two sub-clusters that comprise the East Coast cluster upon which climate predictions and models are based (CSIRO 2015). Thus, the climate change projections discussed herein are focused at the cluster level to better reflect the totality of anticipated change, shifts, and impacts of water-related climate extremes within the study region. Within the East Coast climate analysis cluster, the projections indicate a moderate probability of decreased winter rainfall events, and a strong probability of increased intensity of extreme rainfall events (CSIRO 2015). The hydroclimatic regime along the eastern Australian coast is dominated by several complex climatic mechanisms that occur along annual and decadal timelines (Thyer and Kuczera 2000). Ocean processes strongly affect the hydroclimatic regime where the majority of the population resides, along coastlines. Australia is significantly affected by the El Niño-Southern Oscillation (ENSO), particularly as related to rainfall intensity, duration and frequency as well as droughts (Beer 2014; Thyer and Kuczera 2000). In eastern Australia, a prolonged ENSO event leads to prolonged drought events (Beer 2014). The most common form of flooding is riverine flooding following heavy rainfall events (White et al. 2015). The precipitation map in Fig. 1 highlights the year-over-year difference in precipitation across the country, with data for QLD (northeast) showing the more than 400–1800 mm deviation in excess rainfall for coastal regions of the state (Australian Bureau of Meteorology 2012). The state of QLD is the country’s second largest with a population of about 3.6 million people and is located in the northeastern part of Australia. The majority of the population live along the South Pacific Ocean coastline.

The Canadian study region has a unique hydroclimatic regime that is dominated by cold weather processes including snow accumulation and melt, frozen soils and glacier runoff (Pomeroy et al. 2007). Although snowfall accounts for only about one third of total annual precipitation, snowmelt water represents about 80% of the runoff and recharges soil moisture, groundwater and surface water storage. With the exception of intense summer convective storms, very little summer rainfall runs off. Local prairie water resources are limited and very sensitive to changes in climate and land cover. Few prairie-sourced streams can support substantial water diversion. A seasonal shift towards earlier snowmelt runoff has produced a decline in summer streamflow. A recent analysis of streamflow trends in the Canadian Prairie Provinces (St. Jacques et al. 2014) shows a distinct geographical pattern with significant declines in the west and significant increases in the east. The hydrologic regime is changing from being predominantly snowmelt driven to one that includes mixed rain and snowmelt runoff, with projections indicating an increasingly rainfall-dominated regime. The precipitation map in Fig. 2 supports this projection with higher than normal precipitation rates in evidence in 2011 across the EWE-impacted region. The combined impacts of an earlier start to spring snowmelt, a shift towards a more rainfall-dominated hydrograph, and overall reductions in both total annual and summer flow volumes may lead to significantly higher runoff (flooding potential) in winter, with significant and prolonged drought in summer, interspersed with intense summer convective storms that may produce flooding under drought conditions.

Thus, the projections for rainfall and driving hydrologic forces in each of the study regions denote the need for impact assessments that consider risks of both drier and wetter climatic conditions, possibly simultaneously, as rainfall-driven regimes shift toward higher intensity, but less frequent rainfall events.

### Template Analysis

Government documents from four jurisdictions were collected and assessed per reporting on the outcomes, impacts, and requirements for improved response in subsequent events of 2011 floods in the two selected regions. Government reports on the impacts of the Queensland flood in Australia were produced immediately following the event (Holmes 2011) and later in response to more extensive community impact analysis and review of government responsiveness (Sofronoff 2015). Documents describing outcomes and impacts of the 2011 flood that affected the Canadian Prairie provinces (and northwestern Great Plains in the USA) include three provincial government documents (Alberta Innovates 2014; MB Task Force 2013; WSA 2011) and three American government documents (NDDES 2011; NOAA 2012; USACE 2012). Template analysis was selected for its applicability to analysis of qualitative secondary data contained within these documents and government reports.

In template analysis, a series of themes, or a priori codes, are predetermined either from an expectation of appropriateness for such codes to be contained with the documents given the context of the analysis or specifically on the basis of identifying themes contained within the research question or hypothesis under investigation (Andriotis 2010; Brooks and King 2012; King 2012). Further, there is clear value in the literature, understandings of limitations, and best practices for template analysis methods related to the types of information and communication reports essential for EWE research including environmental, risk management, and structural reviews and governance assessments (King 2012).

Generally, the results of template analysis are grouped by themes and presented in terms of numerical descriptions of occurrence of a priori themes within a given information set. For the research in question, 20 themes were grouped into 4 categories for template analysis. The four categories included (1) proactive management and coordination efforts; (2) disaster management; (3) disaster recovery; and (4) psychological barriers to mitigation community action. The 20 themes were categorized according to communications, community engagement, and both government and community responses to the 2011 floods; themes were populated via keyword and concept searches contained in the government documents. The template was responsive to findings across the full data set, with concepts added or dropped as fit and contextual quality were focused to the research objectives.

## Key Findings and Emerging Patterns

All eight official government reports that included analysis of the scientific (e.g., hydrology, landuse, design), economic (e.g., business, innovation, development), and public service responses to the 2011 floods in SK and QLD were included in the assessment. Of these, two documents focused on the Australia event and six focused on the Prairies event (3 Canadian and 3 American). Based on the a priori themes identified for completion of template analysis on the defined set of community and government post-event reports, themes were grouped into categories as previously described. Both keyword enumeration and contextual evaluation activities were completed on no fewer than 20 concepts grouped across the four a priori themes within the template analysis method.

### Queensland, Australia

Even with Australia’s world-class communication, education, and emergency response procedures, the outcomes and impacts of the Brisbane flood of 2011 clearly identified that the region’s governments, infrastructures and citizens were not prepared to adequately or fully cope with water issues related to flooding (Bohensky and Leitch 2014; Eves and Wilkinson 2014; Harwood et al. 2014; Shepherd and van Vuuren 2014; Towers et al. 2014; Yates and Partridge 2014).

Simpson (2008) describes a natural disaster as a natural event that places “…demands on the system (that) are greater than the capabilities of the community to meet” (p.646). The Queensland Floods Commission of Inquiry’s (QFCI) Interim Report (Holmes 2011) on the 2010-2011 QLD floods highlighted this incapacity, outlining how the flooding began with isolated flooding in low-lying parts of the state in early December 2010, but by mid-January 2011 nearly 78% of the state had reached the classification of being declared a natural disaster. The geographical significance of these floods was noted by comparing the affected landmass to being larger than that of France and Germany combined and including more than 70 towns and cities including the state capital of Brisbane. The flooding was exacerbated by catchments that were pre-saturated followed by prolonged heavy rainfall that created flash flooding conditions and what has been termed an “inland tsunami” (Small 2012).

Under these extreme conditions, 35 people died as they were washed from homes and vehicles in the towns of Toowoomba and Grantham located approximately 150 km inland from Brisbane. The original and a subsequent inquiry into factors leading to this devastation assigned blame to “an unpreventable natural disaster” with an insignificant role being assessed per local infrastructure (including railway embankments), business and industrial activities (such as quarries), or municipal design features (like impervious surfaces and detention ponds) (Sofronoff 2015). Overall, the state experienced impact to more than 2.5 million people with 29,000 homes and businesses being affected in some manner (Holmes 2011).

The QFCI Interim Report (Holmes 2011) further outlines how multijurisdictional organizations collaborated in disaster response and recovery, with over 55,000 registered volunteers in Brisbane and surrounding towns. However, even with warnings and forecasts by the Australian Bureau of Meteorology, decisions were made at a regional level that impacted the severity, impact, and duration of flooding.

### Saskatchewan, Canada

The southern Canadian Prairie provinces and northern USA Great Plains region experienced a 2011 flooding event classified to be the worst in recorded history in terms of both scope and severity. A “perfect storm” of environmental conditions laid the foundation for extreme flooding as mountain meltwaters moved east from Alberta (AB), through Saskatchewan (SK), into North Dakota (ND) and Minnesota (MN, USA) and back to Manitoba (MB, Canada) where the water enters Lake Winnipeg on its way to Hudson Bay and the Arctic Ocean. These conditions included high soil moisture prior to winter freeze-up, elevated groundwater tables, higher than normal winter snowfall, above normal and later than usual combined spring snow and rainfall events, and significant summer rainfall. Combined with severe wind events that created large waves and increased the erosive force of the water being held back by embankments, reservoirs, and transportation systems (roads and highways), these conditions led to widespread severe flooding as well as infrastructure failures, including large cuts through the nation’s highway, the Trans Canada Highway.

In SK, these events began during a technical drought in April during which no rainfall had fallen (Environment Canada 2011). The breakdown of communications and emergency response plans across interprovincial and international boundaries as these events aligned and accumulated through downstream regions led to the flood that was deemed to be of a scope and severity never before experienced in recorded history.

To minimize local impacts, each jurisdiction opened floodways that allowed for higher volume and flows to travel downstream, thus exacerbating the cumulative downstream impacts as the excess water moved from the Rocky Mountains to Hudson Bay via Lake Winnipeg (Alberta Innovates 2014; MB Task Force 2013; NDDES 2011; NOAA 2012; USACE 2012; WSA 2011). Each affected state and province conducted formal reviews of jurisdiction, predictive requirements and appropriateness of response; on the basis of these reviews, there is little evidence of coordination in either post-event assessment or planned responses for future events. Based on this lack of evidence, researchers and community members alike question whether or not lessons have been learned. This lack of coordination further points to the absence of support for community engagement toward improving community resiliency in response to future EWE.

The temporary loss of the Trans Canada Highway in southeastern SK due to erosive forces, and the flooding or purposeful cutting of secondary and tertiary road systems that eliminated these as transportation routes, led to extremely circuitous detours (10 h or more) and reduced community access to resources (fresh groceries) and services (health care). Road systems were cut to release pressure and divert water away from vulnerable downstream communities, farm roads and railway embankments that had served as flood protections failed under hydraulic pressure and erosion, agricultural lands and foraging areas were lost from production and access for at least 2 growing seasons, and many communities were completely isolated from all social services and supports. Across the affected watershed, each province and state reported disaster recovery expenses of more than $1.2 billion (both CDN and USD currency) (Alberta Innovates 2014; MB Task Force 2013; NDDES 2011; NOAA 2012; USACE 2012; WSA 2011).

### Proactive Management & Coordinated Efforts

To fully elucidate the challenges and opportunities involved with the proactive management of physical and socio-economic systems in the wake of an EWE, issues and concepts were coded to identify challenges, successes, and opportunities for improvement related to jurisdiction (including for Canada, the International Joint Commission that supports water management along and across the Canada–USA border); communication; infrastructure design and downstream capacity analysis (i.e., water storage); community resiliency and vulnerability; and community and government department capacity.

Across datasets from Australia and Canada, there was a dearth of information that supported effective implementation of proactive responses and management techniques. The government documents noted that the majority of communities in the affected regions reported having disaster management plans on file, but that many of these were inadequate for the scale and type of flood experienced. One particularly significant finding noted in the post-event reports is that few community plans have they been tested at either the community scale or in concert with proximate community partners (e.g., across an affected watershed or flood plain). In both study regions, the analysis points to a need for scenario planning and mock testing of plans in-community and regionally.

Along the Canada–USA border shared responsibility is coordinated through the IJC and interprovincial bodies (such as the Prairie Provinces Water Board) that were created originally to improve drought management (e.g., reservoir and dam development) but have, of necessity, add flood management to their repertoire. During flood events, decisions tend to be made on the basis of regional hydrologic models that quantify potential cumulative downstream impacts rather than at the more granular community or individual farm or industrial site scale. The post-event government reports from both Canadian and American agencies noted poor communication and coordination of efforts that engaged communities to participate in decision-making. Rather, decisions were made at the provincial and state scale in consultation with communities. In Australia, the interstate communications systems and proactive management plans for EWE are world-class, but functioned in an apparently limited or lacking capacity during the 2011 flood, as evidenced by documented impacts and loss of life and property as a direct result of that EWE. During the 2011 QLD flood, six governmental agencies plus local councils were involved in the coordination, communication of risks and hazards, and responses to dangerous flood waters. Of particular benefit was the amalgamation of agencies within a flood operations centre where experts from each unit collaboratively confirmed weather forecasting, real-time flow gauging, and decision-making processes for operation of dam and reservoir systems, predictive models, and coordination of post-event evaluation of systems. At the height of the flooding, documents show that the internal communications systems appeared to be functioning well and were noted as highly proactive and well-coordinated. External to the flood operations centre, however, the communications with affected publics were seemingly less adequate, as noted in the QFCI report (Holmes 2011).

Both study regions had constructed water diversion and storage facilities to protect communities, lives, and livelihoods. In both study regions there remain questions about the adequacy of these facilities to handle the increasingly frequent, high intensity and high volume floods, and whether or not the communities proximate to such facilities are empowered to appropriately communicate, coordinate, and operate those facilities during EWE. Further unanswered questions focus on the degree of jurisdictional responsibility focusing on coordination and contributions from affected provinces and states as related to unequal impacts and responsibilities from minimal response and concern in upstream reaches of a watershed to potentially disastrous cumulative response and facilities requirements downstream. The challenges associated with design, construction and operation of a collaborative interprovincial and international network of responsive reservoir systems to serve at not only the community and regional scale, but also respond to multi-jurisdictional flood and drought events must be resolved. At a 2004 workshop in SK, key themes related to capacity building emerged, including human, social, economic, and environmental capital (Ramin 2004). The 2011 SK flood and reports detailing inadequacies in managing these complexities, and point to significant failures to plan, coordinate, and communicate across jurisdictions. The reports further point to failures in community engagement and decision-making related to local scale technological and infrastructure response (MB Task Force 2013; NDDES 2011; NOAA 2012; USACE 2012; WSA 2011).

In Australia, interstate collaboration for proactive management and coordination of responses and infrastructure development has resulted in a series of dam and reservoir systems that can, at least partially, mitigate flood impacts through staged operation and cumulative storage capacity (Holmes 2011). Based on the outcomes and reports detailing the efficacy of use and coordination of these staged facilities during the 2011 QLD flood, there is room for improvement to coordinate efforts (Sofronoff 2015; Holmes 2011).

In both Canada and Australia, the results noted in the government reports indicate a relatively strong degree of provincial and state level understanding of the need for flood mitigation facilities, strategies, and decentralized systems. However, based on the outcomes of the flooding at the community and individual level, it was noted that the coordination of those facilities and strategies was not optimized at the community level, resulting in not only unanticipated and unforeseen damages, but also an intensification of resentment regarding decisions to intentionally flood property to avoid downstream devastation and toward upstream jurisdictions who were seen to be passing along a cumulative problem without adequate intervention and capacity (whether physical or social) to manage flood waters.

### Disaster Management

During an extreme water event, several issues must be adequately managed to ensure citizen safety and to both understand and effectively communicate the associated dangers. These include education and communication about entering flood zones without due care and attention (e.g., driving into a flooded area) during an active event, and efforts for maintaining current information about affected and vulnerable buildings (e.g., presence of hazardous materials or child care centres). Information about the latter implies people are cognizant of building codes and municipal zoning (e.g., building in a flood zone, sandbar, or below high-water lines).

The reports comprising the dataset for this research made note of several failures or challenges faced with respect to communicating, responding, and educating various publics in the midst of a disaster. Communication during an EWE is an essential activity not only for the protection of citizens, their property and livelihood, but also for disaster respondents and managers to be able to function in a safe environment not complicated by the actions of well-intentioned private citizens. During a flood-related EWE, the dangers presented by the unnecessary or unknown presence of citizens in a disaster zone creates risks for both the citizens themselves, as well as those responding to an event. During a drought-related EWE, unauthorized or unregulated interventions by private citizens can cause distress to downstream water users whose access may become seriously limited and could possibly even jeopardize a community’s capacity to respond to a non-water event (such as a wildfire). Significant challenges exist between interests of the commons and those of private citizens (Berkes 2009), yet these intersect and are interdependent in terms of how communities can respond to extreme events.

A community’s resiliency and ability to address EWE-related vulnerabilities can be directly tied to its ability to effectively and appropriately respond during a crisis situation. In both Australia and Canada, the post-event government reports consistently point to a general lack of public knowledge and understanding of the dangers associated with both the 2011 extreme flooding events (Sofronoff 2015; Holmes 2011; MB Task Force 2013; NDDES 2011; NOAA 2012; USACE 2012; WSA 2011).

The GFCI (Sofronoff 2015) noted specific causes of the Grantham flood including failures of physical and engineered structures. It highlighted how eyewitness accounts were considered in decision-making, and how landscape amendments affected the impact and extent of flooding across the region. In the final analysis, there are clear indications of a lack of knowledge and understanding from different sections of the general public regarding the functionality of systems and structures, as well as how and where water moves within and across landscapes, despite human interventions to the natural environment (Sofronoff 2015). Of particular note are post-event statements regarding how local police services had to actively limit access to and, several times, remove people (and their vehicles) from dangerously flooded areas in order to ensure public safety while conducting searches for missing persons. It seems the general public tended to not understand the impact and causation of breaches in quarries, railway lines and riverbanks. One odd contribution in the final GFCI (Sofronoff 2015) report notes that it wasn’t within the purview of a key taskforce (prior to the GFCI) to consider the cause of flooding, and thus, there were no lessons learned or recommendations regarding development of more proactive education campaigns to better prepare communities for subsequent EWE.

In the comparable Canadian and USA documents, similar notes pointed to the lack of public understanding about the natural paths, movement, and potential for destruction that waters could have during flooding. These are evident in home- and land-owners’ refusal to evacuate during diversion activities that placed them in the direct path of excessive flood waters. In their post-event report, the NDDES (2011) noted that the involvement of 32 state agencies supporting disaster recovery through a collaborative task force surely contributed to enhanced supports in some areas and increased confusion in others.

In both study regions, minor to serious incidents of people attempting to identify creative ways to leverage insurance policies to replace or repair personal property, such as vehicles or equipment, were documented. In Brisbane, reports of people driving directly and deliberately into flood water to invoke vehicle replacement insurance for new luxury vehicles were documented. Thus, while insurance policies are part of the solution for reducing community and personal vulnerability to EWE, there are also examples of how such systems can be manipulated or abused for perceived personal gain. Generally, however, such incidents point to poor incident communication whereby citizens endanger themselves and others (most notably first responders who are distracted or diverted from other essential activities).

The post-EWE reports point to a need for appropriate management and dissemination of information to and with citizens during a disaster to ensure harmonious and resilient responses that lead to effective recovery.

### Disaster Recovery

How well a community is able to respond to disasters associated with EWE is dependent upon putting into effect a comprehensive and well-considered community-specific disaster response plan. Such plans include having prepared, well-trained response teams and strong leadership in the face of disaster to support recovery of the community and its functions post-event. Disaster recovery in the aftermath of an EWE (or in the midst of an extended and extensive drought) must address the financial, physical, environmental and social costs, including influences of the actuarial and insurance business (e.g., policy coverage, payouts, gaps, and uninsured losses), as well as targeted government assistance, subsidies, and personnel. Recovery also must take into consideration the loss or damage to life, habitat, and infrastructure, as well as diminished environmental capacity and function. From disaster recovery, there is an expectation for intersections with proactive management and continual improvement of community disaster response plans.

From the results of template analysis in the evaluation of disaster response and recovery, inadequacies were noted in many community disaster response plans, particularly those associated with coordination and communication with surrounding jurisdictions. In sparsely populated rural regions, more than seven examples were provided in the two sets of EWE reports of instances where community response did not account for, nor include prior discussion with, upstream and downstream neighbours to include and understand how community-level decision-making would negatively impact the results of upstream decisions and downstream communities’ abilities to respond appropriately.

Of further concern is the rate of response post-event to produce high quality, constructive, and forward-thinking documents available to the public that provide guidance for subsequent EWE management, response, and recovery. Of note, the Australian government produced public documents with extensive recommendations for improvement within 6 months post-event (Holmes 2011) with further analysis, as well as prevention and recovery documentation released after considerable time and public consultation (Sofronoff 2015). In the USA, the reports were much-abbreviated in comparison and, with the exception of North Dakota (NDDES 2011) were released approximately 12 months post-event (NOAA 2012; USACE 2012). However, no follow-up documents are available to demonstrate implementation of disaster recovery plans or guidance. The post-event reviews, evaluations and responses from the responsible agencies in the Canadian Prairie provinces were not publicly released until 24 to 30 months post-EWE (MB Task Force 2013; WSA 2011). Although these documents are significantly more comprehensive and constructive than the comparable documents from the affected American states and responsible agencies, it must be noted that the Australian approach to staging the availability of information and guidance, with reports 6-months and 4 years post-event, is considerably more constructive and responsive to concerns of citizens, industry, and agencies. Such an approach further supports community engagement such that there are immediate lessons learned for discussion and implementation, even in emergency planning if not an actual emergency situation.

The Australian and Canadian study regions have government financial schemes (subsidies and disaster recovery funds) in addition to the insurance policies that may be held by private land and property owners negatively affected by flooding. However, it is increasingly difficult to obtain flood insurance for damage occurring via overland flow, particularly with the increasing frequency of and expenses associated with such events. Drought and flood insurance is available for agricultural producers in terms of crop loss and general infrastructure damage, but these too are becoming more expensive and less inclusive. Regardless, in most cases a combination government support or private insurance provide aid in recovery post-event; the proportions tend to be event-specific as to whether or not the person or the public bears more responsibility. For instance, flooding often occurs either as a result of being directly in the path of flood water or as a result of decisions made by public service experts to divert flood waters (e.g., cut roadways to release hydraulic pressure and divert large flows) away from high-density population zones and areas containing hazardous materials or vulnerable populations. Such financial settlements and payments can offset the cost of disaster recovery, but rarely do they address proactive solutions that enhance or improve previously available technologies and infrastructure have been shown to be inadequate in the face of flooding, for instance.

The reported information, post-event responses and lessons learned about the effects of EWE, are most often hindered by restrictive public policy and insurance policies. These policies also limit the practical application of lessons learned as they give preference to direct replacement of equivalent pre-existing facilities (rather than upgrading) or ecosystem services. Such barriers to change exist due to the design of economic systems and influence both engagement and empowerment of communities to adequately reduce future vulnerability to such extremes.

In Commonwealth jurisdictions, there is a movement toward community health care models that can demonstrate the benefits of an empowered community (Lacey et al. 2015a, b). It is anticipated that similar approaches toward community empowerment for EWE interventions can enhance social fabric within and across communities (in support of improved collaboration, communication and cooperation in regions) and engender a level of value for each other that can reduce a community’s likelihood of simply passing along a problem or exacerbating a problem to downstream neighbours. This premise is supported by data collected in the democratic engagement component of the Canadian Index of Well-Being (Moore et al. 2010), which indicates that Canadians tend to be highly involved in democratic processes and decision-making; essential elements for successful implementation of community empowerment models.

## Conclusions

Within scientific understandings about the projected increase in extreme water events in the two study regions, there is a need to further evaluate and enhance the political, education, and community understandings and responses to these EWE. By leveraging key themes that have emerged from the analyses of government and industry reports, we can identify and evaluate the potential effects, opportunities and challenges that can result from addressing ongoing deficiencies in communication, skills and capacity, and proactive understandings of community involvement and responsibility towards EWE. Suggested reframing of the way we view and communicate both the natural and human factors that create and mitigate EWE can present opportunities to construct more sustainable community engagement. This engagement may promote emancipation whereby citizens and local community leaders have a voice in the planning and mitigation processes around preparing their communities for EWE. Processes that recognize that science and technology alone cannot prevent or respond to all of the potential negative impacts of EWE.
